# Around the Hospitals

**Published:** 1893-04-15

**Authors:** 


### Around the Hospitals.

Notice to the Managers of Institutions.—Special space is now reserved for the insertion of notices of hospital meetings and festivals. The Editor requests that all notices may reach The Hospital Office, 428, Strand, at least one week before the date of each meeting.

**University College Hospital.**—The annual meeting of this hospital was held on the 24th ult. An excellent report of the year's work was given. Especial attention was drawn to the decrease of mortalities after operations. It is a matter of congratulation that the heavy debt on the institution has diminished, and that subscriptions and donations alike show an increase. The People's Contribution Fund amounted to £405. Towards the much-desired extension £27,000 has now been collected. It is proposed to close the hospital in the autumn for repairs.

**St. Mary's Hospital** held its forty-second annual meeting on the 24th ult., which was presided over by Mr. J. Derby Alcroft, J.P. During the meeting Mr. Paris Singer telegraphed a donation of £100. Attention was especially drawn to the inadequacy of reliable income. It was pointed out that the hospital is situated in a wealthy vicinity. The hospital has been at a disadvantage, secluded as it has been from view by surrounding buildings. the Clarence Memorial Wing, which will bring it into prominence, should effect a change for the better. Towards this extension, the foundation-stone of which was laid by the Prince of Wales in December, the sum of £13,015 has been added during the present year. In this direction, and in the acquisition of £10,557 through legacies, the hospital has been fortunate. Unless, however, another £10,000 is forthcoming during the present year the maintenance of the hospital, requiring an income of £23,000, will be a matter of serious difficulty, the reliable income amounting to £13,415. The rating of the hospital is now increased to £229, a serious item of expenditure. The work of the hospital continues to increase, and the valuable addition of a convalescent home, rent free, by Mr. F. Wilder has been made during the past year.

**The London Temperance Hospital** is fortunate in having passed through a year of prosperity, and in possessing many generous friends. The accounts show a balance in hand. Baron Hirsch contributed £700 and the Duke of Westminster £500, in addition to his annual subscription of £100. Mr. J. E. Taylor has provided electric lighting throughout the hospital. The present wants of the hospital are, a Turkish bath and Convalescent Fund.

**The Metropolitan Hospital.**—It is always greatly to be regretted when a hospital is forced to reduce the number of beds available to patients. The Metropolitan Hospital has passed through very adverse times during the past year, and this has necessitated the announcement that twenty-four beds have had to be closed. This is especially unfortunate, as the calls on the hospital have been greater han ever, and many urgent cases are now of necessity refused admission. The adverse balance is £5,531, and to pay ordinary expenses the committee has decided to raise a further mortgage of £4,000. It is to be hoped that funds will be forthcoming to reopen the Louise Ward, and that the forthcoming festival will greatly increase the resources of the hospital.

